# Hospital-based evaluation of palliative care among patients with advanced cervical cancer: a cross-sectional study

**DOI:** 10.1186/s12904-022-01030-2

**Published:** 2022-08-03

**Authors:** Tolcha Kebebew, Annah Mosalo, Azwihangwisi Helen Mavhandu-Mudzusi

**Affiliations:** 1 Ethiopia Regional Learning Centre, University of South Africa, Addis Ababa, Ethiopia; 2 Department of Health Studies, University of South Africa, Pretoria, South Africa

**Keywords:** Cervical cancer, Home care, Hospital care, Pain, Social care, Supportive care

## Abstract

**Background:**

Palliative care is among the standards of care in cancer treatment that should be provided to those in need within the existing healthcare system. In Ethiopia, patients with cervical cancer experience a long wait for curative radiotherapy, while the level of palliative care delivery is unknown. In this study, we aimed to evaluate the practice of palliative care among women diagnosed with advanced cervical cancer.

**Methods:**

A hospital-based cross-sectional study was conducted. Using a structured questionnaire, face-to-face interviews were made with randomly selected patients with advanced cervical cancer. Information on patient characteristics, medical records, and knowledge, attitude and practice of palliative care was captured, analysed, and presented. Data collection was conducted following ethical standards after obtaining approval from the hospital.

**Results:**

A total of 385 patients were interviewed, most of whom were over 50 years and illiterate. The patients had poor knowledge regarding comprehensive palliative care, a good attitude, and poor practices. Most patients either do not know about palliative care or consider it solely as a pain treatment. The patients expressed a good attitude towards palliative care; however, their attitude towards talking about suffering, death, and dying was poor. Almost all patients have received some form of palliative care. However, poor pain control, inadequate education and counselling, and poor social, economic, and spiritual supports were documented.

**Conclusions:**

Patients with advanced cervical cancer expressed a good attitude but had poor knowledge and practice of comprehensive palliative care. The palliative care delivery needs to address the communication, psychosocial, economic, and spiritual components of the comprehensive palliative care.

## Background

Cervical cancer is one of the major public health problems globally, and the fourth most common cancer among women worldwide [1]. In the developing world, cancers of the cervix and breast are the two most common types among women. Due to low vaccination coverage against Human Papilloma Virus and poor screening uptake, cervical cancer has not been controlled in developing countries [2, 3].

High morbidity and mortality rates from cervical cancer are reported in Ethiopia, with incidence and mortality rates of 21.5 and 16.0 per 100,000 population in 2020 [2]. Although cervical cancer is curable at an early stage, stage IIA or less, according to the International Federation for Gynaecology and Obstetrics [4]), the prognosis at advanced stage, even after sophisticated curative treatment, is poor [5, 6]. In Ethiopia, most patients are diagnosed at an advanced stage [7–9], at which point the patients suffer from the burden of devastating symptoms [5, 10–12]. Many patients attending a tertiary hospital in Addis Ababa were at an advanced stage [8, 9, 13, 14]. At an advanced stage, patients benefit more from palliative care, aiming at alleviating symptoms and problems associated with the disease, than from expensive and long-awaited curative treatment [5, 12, 15, 16].

Palliative care is comprehensive care provided to patients with life-threatening disease conditions to improve the quality of life of the patients, caregivers, and their families [5, 16]. It is a multi-disciplinary intervention to reduce suffering among patients with advanced, serious or life-threatening diseases, such as cervical cancer. Palliative care includes treatment of pain, management of symptoms and problems, and emotional, socio-economic, psychological and spiritual supports. It aims to alleviate patient and family stresses, and improve or maintain the best possible quality of life.

Due to the increasing trend in non-communicable diseases and aged population, over 40 million people require palliative care globally, most of whom are from developing countries, and only 14% of those in need receive it [16]. Palliative care is among the standards of care in cancer treatment [17]. In addition, the 67^th^ world health assembly of the World Health Organization has endorsed a recommendation that enables the integration of palliative care into the public health service delivery system. This recommendation came after recognising palliative care as a fundamental human right. Member states of the World Health Organization, including Ethiopia, are urged to develop, strengthen and implement comprehensive, evidence-based, and cost-effective palliative care services at all levels of the existing healthcare system [18]. The government of Ethiopia has included palliative care among the priority areas in the Health Sector Transformation Plan 2020–2025 [19]; however, the country’s provision of palliative care is limited [20].

While there should have been a priority to palliative care over curative treatment, patients attending the tertiary hospitals did not benefit from comprehensive palliative care. Most patients diagnosed with cervical cancer suffer from various symptoms, including pain, loss of appetite, anxiety, confusion, and vaginal discharge [21]. In addition, the patients face psychosocial and economic stresses resulting from the disease and its treatment. Early initiation of palliative care can help relieve these symptoms and problems and enhance the quality of life of the patients, caregivers, and families. There are no empirical studies in Ethiopia on t he status of palliative care among patients with cervical cancer.

This study evaluated the levels of comprehensive palliative care provided to patients diagnosed with advanced cervical cancer receiving treatment at a tertiary hospital in Ethiopia. We also assessed the patients’ knowled ge and attitude towards palliative care. 

## Methods 

A hospital-based cross-sectional study was conducted in a radiotherapy centre of a specialised hospital in Ethiopia from January to June 2019. The hospital is the only centre that provides comprehensive cancer treatment with radiotherapy, chemotherapy, and surgery. Patients diagnosed with cervical cancer, mainly those at an advanced stage, are referred to the centre for evaluation and treatment. This study is part of the principal author’s thesis a t the University of South Africa, with the methodology section published elsewhere [21]. 

The sample size for this study was calculated using formulae for a single population proportion, using the prevalence of 50%, level of confidence of 95%, margin of error of 5%, and 5% additional for non-response, which yielded 404. The study participants were selected using random sampling. Patients with cervical cancer at the stages IIB-IVB attending the centre were identified and included in the study. Other inclusion criteria were patient consciousness, communication ability, and willingness to give informed consent. Patients without established cancer stages were excluded.

A structured and pre-tested questionnaire was designed to collect information, including patient characteristics, treatment information, knowledge, attitude, and practice of palliative care. The knowledge questions assessed the patient’s awareness of palliative care and its components. The tool for measuring patients’ attitudes towards palliative care was adapted from previous studies [22, 23] and the Palliative Care Evaluation Tool Kit [24]. It included 15 five-level Likert Scale items. Responses ranged from 1 referring to the poorest attitude, denoted by 'strongly disagree', to 2 for 'disagree', 3 for 'neutral', 4 for 'agree', and 5 for 'strongly agree' representing the best attitude. The researchers developed items used to measure pain management, symptom management, psychosocial care, economic support, and spiritual care. The data collection tool was translated into and used in Amharic, the local language.

Trained hospital nurses and the principal researcher collected the data. Each day, two to three patients per data collector were selected using a simple random sampling technique. The completed questionnaires were reviewed for consistency and missing variables before data entry. The data entry and cleaning were conducted using CS Pro software. The final dataset was transferred to Stata 12® for analysis.

Findings from the data analysis were presented in n umbers and percentages for palliative care knowledge and practices. On the other hand, proportions, means and standard deviations were computed for the attitude scores. Items representing negative attitudes were reversed before computing aggregate scores. Responses 4 and 5 (agree or strongly agree) were recoded into “yes”, which represented the proportion of the respondents who agreed with the items, while the other responses (1, 2 and 3) were recoded into “no”, representing respondents who disagreed with the items. The aggregate score for the scale and sub-scales was computed and reported by calculating mean and standard deviation of the items that contributed to the scale or subscales. 

## Results

### Characteristics of respondents

Successful interviews were conducted among 385 (94% of the 404 samples) patients diagnosed with advanced cervical cancer. The age ranged from 20 to 80 years, the average being 52 years. Most study participants were illiterate, married, and with a low monthly income (below US$50), (Table 1). Half of the patients were at stage II-III, whereas the other half were at stage IV. Over half of the patients took palliative radiotherapy, and about one-third took curative radiotherapy and chemotherapy. This information has also been published elsewhere [21].

**Table 1:** Socio-demographic and treatment information of patients with advanced cervical cancer, 2019

Patient Characteristics	Number	Percentage
*Age (Years)*		
Below 50	147	38.2
50 and above	238	61.8
*Educational status*		
Illiterate	243	63.1
Literate	142	36.9
*Marital status*		
Currently in marriage	240	62.3
Currently not in marriage^a^	145	37.7
*Occupation*		
No job	123	32.0
Housewife	81	21.0
Farming	72	18.7
Others^b^	109	28.3
*Average monthly income (in USD)*		
Below 50.00	324	84.2
50.00 or more	61	15.8
*Cancer stage*		
IIB	90	23.4
III (A or B)	105	27.3
IVA	162	42.1
IVB	28	7.3
*Treatment modality*^c^		
Surgery	39	10.1
Chemotherapy	135	35.1
Therapeutic radiotherapy	139	36.1
Palliative radiotherapy	216	56.1

^a^includes single or dissolved marriage;

^b^Others include retirees, employees, petty traders and daily labourers;

^c^multiple responses possible; USD-United States Dollar. Reproduced from authors’ publication, *Kebebew *et al*. 2021* [21]

### Palliative care knowledge

Approximately one quarter (*n* = 100; 26.0%) could explain the term “*palliative care*”, locally translated as */yämastagäsha enkbkabä/*, meaning “relieving or easing patient symptoms without curing the underlying disease”. Those who had knowledge about palliative care explained that it is pain control, treatment of symptoms, education, counselling, and family and social support (Table 2). Major sources of information on palliative care were hospitals (*n* = 96; 24.9%), followed by the community (*n* = 14; 3.6%), support groups (*n* = 10; 2.6%) and health centres (*n* = 9; 2.3%). Other sources of information were private health institutions, health posts, and the mass media. However, 241 (62.6%) explained that it is all about pain relief, and 11.4% (*n* = 44) did not know about palliative care (Table 2).

**Table 2:** Knowledge of patients with advanced cervical cancer regarding palliative care, 2019

	**Number**	**Percentage**
**Palliative care knowledge (*n* = 385)**		
Know palliative care	100	26.0
Explain it as pain control	241	62.6
Do not know	44	11.4
**Knowledge of palliative care (*n* = 385)**^a^		
Pain treatment	338	87.8
Symptom control	79	20.5
Counselling	78	20.3
Education	55	14.3
Family support	50	13.0
Social support	24	6.2
Financial support	16	4.2
Preparing for death	8	2.1
**Source of information (*n* = 100)**^a^		
Hospital	96	96.0
Health centre	9	9.0
Health post	5	5.0
Private health facilities	7	7.0
Home/Community	14	14.0
Support groups	10	10.0
Mass media, books	3	3.0

^a^Sum of percentages is not 100% because of multiple response options

### Attitude towards palliative care 

Fifteen five-scale items measured attitude towards the palliative care services, coded from 1 (strongly disagree) up to 5 (strongly agree). The mean and standard deviation scores were 3.4 ± 0.5, which is above 3.0, the mid-point. The majority (*n* = 310; 80.5%) scored above the mid-point in the overall score (Table 3). The sub-scale scores also showed an overall positive attitude towards palliative care, average ranging from 3.1 ± 0.6 in attitude towards suffering and death to 3.3 ± 0.9 in attitude towards physicians, 3.5 ± 0.8 in attitude towards palliative care services, and 3.6 ± 1.0 in attitude towards the use of morphine or other anti-pain drugs.

**Table 3:** Attitude of patients with advanced cervical cancer towards palliative care, 2019

Attitude items	Agree N, %		Mean^a^	SD
**Attitude towards suffering during the process of death**				
Dying patients have little control over their treatment (N)	229	59.5	3.1	1.7
Suffering is part of dying (N)	165	42.9	2.6	1.6
Dying patients have the right to be free of suffering	260	67.5	3.8	1.5
Talking about death can make people lose hope (N)	168	43.6	2.6	1.6
Losing hope makes people die sooner	231	60.0	3.6	1.5
***Sub-scale aggregate***	***252***	***65.5***^b^	***3.1***	***0.6***
**Attitude towards treatment with morphine and anti-pain drugs**				
Treating dying patients with morphine or other anti-pain causes addiction (N)	322	83.6	3.6	1.3
Morphine or other anti-pain is offered only when there is nothing more that can be done (N)	314	81.6	3.5	1.2
Morphine or other anti-pain can make death occur sooner (N)	332	86.2	3.8	1.3
***Sub-scale aggregate***	***334***	***86.8***^b^	***3.6***	***1.0***
**Attitude towards palliative care services**				
It is better to die at home than in a hospital	155	40.3	2.8	1.7
Receiving palliative care means patients are giving up on living (N)	328	85.2	4.0	1.3
Palliative care can make patients feel better	215	55.8	3.5	1.5
Palliative care can make patients live longer	216	56.1	3.5	1.5
Palliative care is offered when nothing more can be done (N)	319	82.9	3.9	1.3
***Sub-scale aggregate***	***291***	***75.6***^b^	***3.5***	***0.8***
**Attitude towards palliative care physicians**				
Most physicians know how to treat pain	265	68.8	3.8	1.4
Caring for dying patients causes stress to doctors (N)	187	48.6	2.7	1.6
***Sub-scale aggregate***	***316***	***82.1***^b^	***3.3***	***0.9***
***Overall score***	***310***	***80.5***^b^	***3.4***	***0.5***

(N) Items with a negative attitude towards palliative care; values for these items were reversed before analysis.

^a^ Negative items were recoded into reverse order before computing; scores range from 1 to 5; 1 referring to very poor attitude, and 5 to very good attitudes towards palliative care;

^b^ Include proportion (%) of those who scored above mid-point (3.0) in the sub-scales

Most of the patients had a positive attitude towards receiving palliative care services. The majority, 328 (85.2%), did not agree with the statement, “receiving palliative care means patients are giving up on living.” Attitude towards receiving morphine and analgesic drugs was also good. A high proportion, 322 (83.6%), did not agree with the statement, “treating dying patients with morphine or other anti-pain causes addiction”, whereas 314 (81.6%) disagreed with “morphine or another anti-pain is offered only when there is nothing more that can be done.” 

**Fig. 1:**
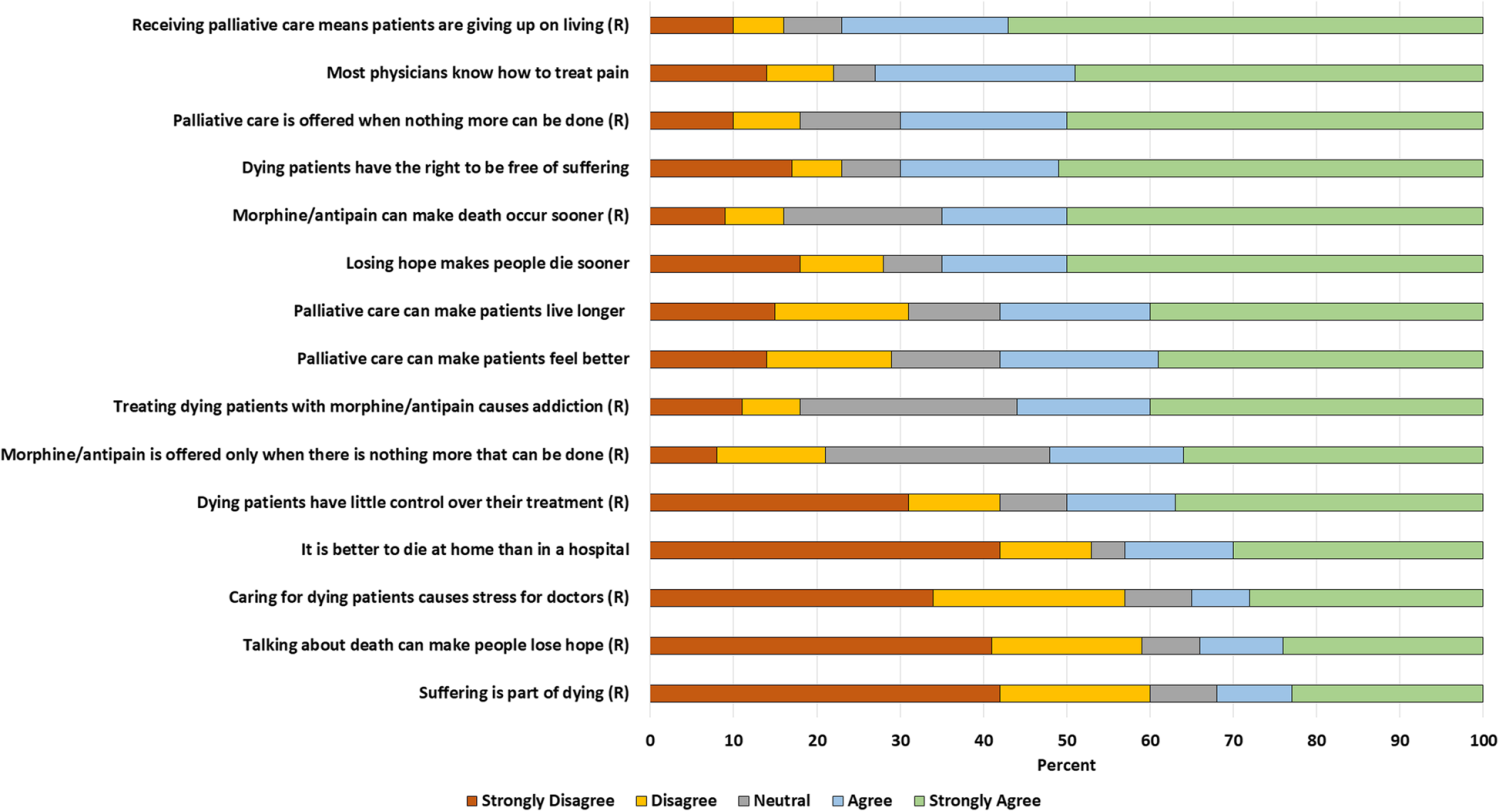
Attitude towards palliative care among patients with advanced cervical cancer, 2019. Note: Responses for negative items denoted by “(R)” were reversed by exchanging agree with disagree and strongly agree with strongly disagree

Over half of the participants had a worrying attitude towards talking about death and the process of dying; 168 (43.6%) did not agree with “talking about death makes people lose hope”, while 165 (43.9%) disagreed with the “suffering is part of dying.” Attitude towards pallia tive care physicians was also poor; 187 (48.6%) disagreed with the statement, “caring for dying patients causes stress to doctors”, while about two-thirds (*n* = 265; 68.8%) agreed with “most physicians know how to treat pain.” Table 3 and Fig. 1 present percentage agreement, aggregate scores, and sub-scale scores of the 15 attitude items.

### Palliative care practices

Almost all patients had received some form of palliative care, consisting of physical, psychological and mental healthcare and social and economic support. However, the level of comprehensive palliative care, including pain control and relief from symptoms and problems was poor.

#### Physical health care 

Nearly all patients (*n* = 372, 96.6%) responded that they had ever experienced pain, and most of them (*n* = 336; 90.3%) received treatment with anti-pain drugs (Table 4). Almost all prescriptions of analgesics were made by physicians (*n* = 327; 97.0%) at hospitals (*n* = 318; 94.6%). However, only about two-thirds (*n* = 189; 56.3%) had been “fairly” or “completely” relieved of pain. The two commonly used drugs for pain control were tramadol (54.5%) and morphine (36.9%). The use of non-steroidal anti-inflammatory drugs, such as diclofenac or ibuprofen, was minimal (*n* = 20; 6.0%).

**Table 4:** Palliative care practices among patients with advanced cervical cancer, 2019

Type of palliative care received^a^	Number	Percentage
Home care by family/caregiver	374	97.1
Treated for pain (*n* = 372)	336	90.3
Fairly or completely controlled pain (*n* = 336)	189	56.3
Treatment for vaginal discharge (*n* = 347)	320	92.2
Treatment for vaginal bleeding (*n* = 322)	304	94.4
Financial support	134	34.8
Advice or counselling by religious people	127	33.0
Home visits by religious people/groups	102	26.5
Education regarding cervical cancer	74	19.2
Advice or counselling by health workers	76	19.7
Provision of educational material	86	22.3
Home visits by health workers/support groups	29	7.5

^a^The denominator is 385 unless specified

The other symptoms for which the patients received treatment were vaginal bleeding and discharge. The majority, 92.2% (320 of 347) and 94.4% (304 of 322) of patients who ever experienced vaginal discharge and bleeding, received treatment. However, the patients reported not receiving treatment for other symptoms, such as poor appetite, weakness, and constipation. 

#### Psychological, spiritual and mental care

A limited number of patients received education and counselling as part of psychological or mental healthcare. Only 76 (19.7%) received counselling at the hospital or elsewhere (Table 4). Similarly, a limited number of patients (*n* = 127; 33.0%) received counselling from spiritual persons, out of which 102 (26.5%) were visited at home. Less than one-fifth (*n* = 74; 19.2%) received education about cervical cancer, including palliative care, and 86 (22.3%) were supplied with educational materials regarding cancer.

#### Socio-economic support 

About one-third of the patients (*n* = 134; 34.8%) received financial support in cash or kind (Table 4). Most of them (*n* = 112, 83.6%) received the support from family and relatives; 27 (20.1%) received support from neighbours, while only 8 (6.0%) received support from social support organisations or volunteers. Almost all patients (*n* = 374; 97.4%) received home care from caregivers, who are mostly family members or friends. The caregivers supported the patients by assisting them with mobility, helping them during visits to health facilities and religious places, cooking, showering, and cleaning cloths and homes. Patients ever visited at home by trained personnel from health facilities or support organisations were minimal **–** only 29 (7.5%).

## Discussions

This study aimed to investigate the palliative care delivery to patients with advanced cervical cancer at a specialised tertiary hospital in Ethiopia. The study identified poor knowledge of comprehensive palliative care. It also revealed that the patients had received some form of palliative care; however, it was non-comprehensive and inadequate. The level of pain and symptoms control, mental care, socio-economic assistance and spiritual support was minimal. In contrast, the patients reflected a positive attitude towards palliative care.

There is no familiar terminology in the local language, Amharic, to assess the knowledge regarding palliative care. In this study, palliative care was translated as “care to relieve symptoms”, translated into Amharic as  , */yämastagäsha enkbkabä/*. About a quarter of the patients (26%) knew palliative care as a comprehensive pain and symptom management and socio-economic and psychological support. However, the majority (63%) defined palliative care as a pain treatment. A study conducted in the same setting reported that 62% of the patients diagnosed with cancer have some knowledge about palliative care [20]. Illiteracy could have contributed to the poor knowledge. 

Pain control was the commonly known type of palliative care, whereas symptom control, counselling, and education were understood among less than a quarter of patients. Spiritual, social and economic support, and bereavement care were almost unknown. Only 2% considered preparing for death as palliative care. In Ethiopia, there are cultural and religion-related sensitivities when disclosing the reality to the patients and families; consequently, physicians or nurses refrain from talking about the enduring nature of the disease, dying and preparing for death [25].

Knowledge about palliative care varies with different factors, including educational status, age, gender, palliative care training, caregiver experience, and profession [26]. A study conducted in Ecuador showed that 79% of the general population had heard about palliative care; the knowledge varied by education, palliative care training, health-related occupation, and gender [27]. However, a study on the general population of Northern Ireland showed that only 20% knew the accurate term, “palliative care” [26]. Similarly, only 29% of adults in the United States expressed good knowledge regarding palliative care [28]. In addition, only 29% of the patients reported good palliative care knowledge in Zimbabwe [29] and Saudi Arabia [30]. 

This study documented a positive attitude towards palliative care. The four categories of attitude assessment tool were attitude towards suffering during the death process, perception regarding anti-pain drugs, attitude towards palliativ e car e service s, and feelings towards palliative care physicians . A relatively poorer attitude was found in attitude towards talking about death and the process of dying. It has bee n show n that 56% of the participants reported that talking about death and the process of dying made them lose hope. The finding is high compared to the study done in the Un ited States in 2013, where only 20% had such an attitude [23]. In this study, a high proportion of the respondents (57%) accepted suffering as part of dying. In contrast, o nly 28% agreed with this attitude in the study conducted in the United States [23].

Attitude towards treatment with morphine and other anti-pain drugs was good. More than 80% of the respondents had a positive attitude; for example, 84% of the participants disagreed that treating patients with morphine or other anti-pain causes addiction. A similar proportion (87%) disagreed that it causes addiction in the study conducted in the United States [23]; however, a higher proportion of Canadian patients (42%) felt it could cause addiction, and 54% felt that morphine could make the patient die sooner [22]. In this study, more than half (56%) agreed that “morphine and other analgesics could make patients feel better and live longer.” However, a higher proportion (82%) of the United States [23] and Canadian (94%) patients [22] felt that the analgesics could make them feel better.

Most of the respondents (97%) had received home care from caregivers. In addition, about 90% of the patients had ever received treatment for the concerning symptoms, including pain, vaginal bleeding, or vaginal discharge. This number is higher than a study done in the same setting in 2014, where 69% of the patients received palliative care services [20]. However, control of the symptoms, including pain, was low.

Among the 90% of patients who had experienced pain, about 11% did not receive any analgesics. Only 56% of patients who received anti-pain drugs controlled the pain. This finding aligns with studies reporting that most physicians and nurses in Ethiopia were reluctant to treat pain [31, 32]. In a study done in the same setting, 93% of cancer patients experienced pain, and 42% had no treatment. Among those treated, 44% received inadequate pain management and inappropriate drugs [33]. Knowledge among healthcare workers could influence the treatment of pain. A survey conducted by the Ministry of Health and partners in Ethiopia showed that only 30% of health workers could conduct correct pain assessments, and 27% did not know the contraindications of anti-pain drugs [32]. Another study in Ethiopia showed that 90% of patients with chronic diseases experienced pain, but only 24% received anti-pain prescriptions [34].

This study identified that the psychological, socio-economical and spiritual component of palliative care was poor among patients with advanced cervical cancer. Only 20% of the respondents in this study received counselling at the centre or in hospitals elsewhere, and a similar proportion received education regarding cervical cancer. About 30% of them received counselling by spiritual persons. In contrast, a study conducted in the same setting showed that a higher proportion (45%) of the patients with cancer received counselling within a year of follow-up [20]. 

This study has some limitations. Firstly, it was conducted among patients visiting a tertiary hospital. Although the hospital is the only place to access patients with advanced cervical cancer, the study excluded patients who died before appropriate diagnosis or those who did not attend the centre. Secondly, the palliative care assessment was limited to patients with advanced-stage, excluding the care provided at the early stage, end-of-life, and bereavement time.

## Conclusions

This study revealed that patients with advanced cervical cancer in Ethiopia had poor knowledge regarding comprehensive palliative care. Awareness regarding psychosocial, economic and spiritual support was low. However, a relatively better attitude towards palliative care services was identified. Similarly, most patients with advanced cervical cancer did not receive mental, social, economic and spiritual supports.

Awareness-raising interventions are recommended to improve the patients’ knowledge regarding palliative care. These interventions must address branding the palliative care term in the local languages, improving communications about dying and the process of death, and appropriate use of strong analgesics for cancer patients. Strengthening comprehensive palliative care at all levels is also recommended. More specifically, tertiary hospitals need to avail comprehensive palliative care that encompasses psychosocial, economic and spiritual support to patients in need. 

## Data Availability

All data analysed during the current study are included in this published article. However, this study is part of the principal author’s PhD project at the University of South Africa. The raw dataset analysed in this study is available from the corresponding author upon reasonable request and after the university grants permission.
